# Menstrual health and hygiene among Indigenous Australian girls and women: barriers and opportunities

**DOI:** 10.1186/s12905-019-0846-7

**Published:** 2019-11-27

**Authors:** Emily Krusz, Nina Hall, Dani J. Barrington, Sandra Creamer, Wendy Anders, Minnie King, Helen Martin, Julie Hennegan

**Affiliations:** 1 The University of Queensland School of Public Health, Herston Road, Herston, QLD 4006 Australia; 2 University of Leeds School of Civil Engineering, Woodhouse Lane, Leeds, West Yorkshire LS2 9JT UK; 3 National Aboriginal and Torres Strait Islander Women’s Alliance, Canberra, ACT Australia; 4 National Aboriginal and Torres Strait Islander Women’s Alliance, Canberra, ACT Australia; 5 Women on Country, Weipa, QLD 4874 Australia; 6 Women on Country, Weipa, NT 4874 Australia; 7 Department of Environmental Health & Engineering, Johns Hopkins Bloomberg School of Public Health, The Water Institute, 615 N Wolfe St, Baltimore, MD 21205 USA

**Keywords:** Menstrual hygiene, Menstrual health, Participatory action research, Australia, Commentary, Indigenous Australians, Aboriginal and Torres Strait Islander Peoples

## Abstract

Health inequities inhibit global development and achievement of the Sustainable Development Goals. One gendered health area, Menstrual Health & Hygiene (MHH), has received increasing attention in Low- and Middle-Income Countries as a barrier to health, wellbeing, and gender equity. Recent anecdotal evidence in Australia highlights that MHH also present challenges to High Income Countries, particularly among underrepresented populations, such as Indigenous Australian peoples, people from low socio-economic backgrounds, or communities that are remotely located. In this article, we chart the emergence of attention to MHH in the Australian context and highlight key considerations for the conduct of research with Aboriginal and Torres Strait Islander Peoples within the culturally- and gender-sensitive area of MHH. Further we draw on insights offered by a partnership between female Aboriginal and Torres Strait Islander leaders, NGO stakeholders, and non-Indigenous researchers. Through a convening (yarning circle) held in March 2018, the group identified multiple socioecological considerations for MHH research and practice, including: affordability and access to menstrual products, barriers to knowledge and culturally sensitive education, infrastructure and supply chain challenges, and the necessity of Indigenous-led research and community-driven data collection methods in addressing the sensitive topic. We draw together these insights to develop recommendations for future research, advocacy, and action in Australia.

## Background

Menstruation is an under-acknowledged challenge that impacts the health and wellbeing of women and girls as well as their broader communities [1, 2]. Despite representing over one quarter of the global population, women and girls of reproductive age often manage their monthly menstruation ill-equipped and in secrecy [2, 3]. Barriers include restricted access to materials to collect menstrual blood, and various water, sanitation and hygiene (WASH) resources in their homes, schools, and workplaces [4, 5]. Resource limitations are often compounded by social and cultural norms that discourage open discussion of menstruation [3, 6]. Generations of girls and women have internalised taboos, secrecy, and shame about the natural processes of their bodies, causing health information about menstruation to either come too late (after menarche—a person’s first period), incompletely, inaccurately, or not at all [2, 4, 7, 8].

Unmet menstrual health and hygiene (MHH) needs have received increased attention in Low- and Middle-Income Countries (LMICs) over the past decade, particularly among school-aged girls [9, 10]. Though evidence is lacking, resource (un)availability and sociocultural environments are believed to impact on girls’ self-esteem, sexual and reproductive health, and school attendance, all of which may affect health outcomes over a woman’s life course [3, 6, 9, 11]. While MHH within LMICs garners worthy attention, stigma and taboo also pervade menstrual experiences within High Income Countries (HICs). More broadly, significant WASH, resource and health-related gender gaps remain among underrepresented communities within HICs [12], particularly those living in low-socioeconomic or remote regions, and due to histories of colonisation and ongoing marginalisation, some Indigenous populations [13, 14].

This article provides perspectives on the challenges of MHH for Indigenous Australian girls and women in urban, rural and remote communities (the authors acknowledge the heterogeneity of Aboriginal and/or Torres Strait Islander Peoples^1^ despite also using the broader term ‘Indigenous Australian’ to refer to Australia’s First Nations Peoples). It describes the emergence of media, policy and research attention to MHH in Australia and critical considerations for the evolution of research on this topic with Indigenous Australian peoples, as well as describing the first steps of an ongoing collaboration between Indigenous and non-Indigenous co-researchers to identify and address MHH challenges for Indigenous Australian girls and women. Drawing together international research, national attention and insights from the convened group, this article concludes with recommendations for research priorities, methods, advocacy and action aiming to identify and address the barriers Indigenous Australian women and girls may face when menstruating.

## Menstrual health and hygiene in Australia

The United Nations’ (UN’s) Sustainable Development Goals (SDGs) include targets for health and wellbeing (SDG 3.7), gender equity (SGD 5.6), and sanitation and hygiene initiatives that provide “special attention to the needs of women and girls” (SDG 6.2) [1]. The SDGs are applicable to all countries (unlike the Millennium Development Goals, which were targeted at improvements in LMICs), yet attention to MHH in HICs has been lacking. Building an evidence-base from which to design interventions in HICs would facilitate equitable opportunities for girls to better prepare for menarche (i.e. onset of menstruation), as well as allow women to better understand biological functioning, identify menstrual-related or vaginal bleeding conditions such as endometriosis, explore various hygiene methods, and participate in shifting social attitudes toward menstruation [2, 7, 9, 18, 19].

Growing evidence and action around MHH worldwide (https://mhhub.org/) as well as emerging qualitative research and anecdotal evidence domestically [20–22] suggest that in Australia there is a need to improve MHH as part of women’s right to experience menstruation without detraction from engaging in full societal participation [23]. The recent removal of Australia’s Goods and Services Tax on menstrual hygiene products is an example of how policy has begun to address inequitable financial penalties that target those who menstruate [24]. However, financial barriers are just one of many factors that contribute to managing menstruation, and despite mobilisation of government and non-governmental responses in the Australian space, there has been a limited research response [2]. The anecdotal reports and growing interest in this issue are timely, but there is a need for evidence to direct effective policy and practice.

Understanding the current state of MHH is a first step in ensuring menstrual equity for all Australian women and girls, particularly in a number of Indigenous Australian communities where assistance in addressing barriers has been requested by Indigenous women leaders of health researchers, for example [25]. Global research and practice suggests that positive menstrual experiences should include having high quality information regarding basic biological needs, access to WASH infrastructure and resources to support menstrual hygiene, and elimination of stigma and taboos that represent an ongoing threat to gender equity as women’s basic bodily processes are marginalised and inhibited as outlined in UN Human Rights Council Resolution A/HRC/39/L.11 [8]. Though this article primarily highlights opportunities for improving MHH among Indigenous Australian communities, it is likely that MHH needs among multiple Australian populations, such as people from low socioeconomic backgrounds, those experiencing homelessness, or those living with disabilities, are also overlooked [22, 26, 27]. Menstrual stigma also impacts women more broadly through the marginalisation of women’s health concerns from menstrual disorders to menopause, which have been highlighted by others and extend beyond the scope of this article [19, 28].

## Conducting health research with Aboriginal and Torres Strait Islander peoples

The diverse communities that comprise Australia’s Aboriginal and Torres Strait Islander Peoples are regarded as the longest living continuous cultures in the world [29–31]. Relatively recent European colonisation of Australia has had repercussive effects that are still visible and ongoing today, and contribute to a deficit-based dialogue regarding Indigenous Australian health [32]. In paternalistic attempts to address the disproportionate health outcomes of Aboriginal and Torres Strait Islander peoples, Western health researchers have often conducted research *on* Indigenous Australian individuals and communities rather than working *with* and being led by Indigenous Australian peoples. These approaches have historically failed to acknowledge the value of non-Western ‘ways of knowing’ (i.e. frameworks that dictate how knowledge can be derived) as well as more holistic definitions of health that incorporate cultural and historical factors [29, 31]. Health research that is harmful or not reciprocally beneficial has further frayed Indigenous Australian peoples’ trust of colonial Australian institutions and non-Indigenous researchers [33, 34]. To move away from exploitative scientific practice, non-Indigenous people who have been invited to partner in Indigenous health research must engage in critically reflexive, participatory practice [29, 31, 35–38]. This approach ideally acknowledges the unique strengths of each particular Indigenous Australian person and community and develops customized programs that move away from largely unsuccessful one-size-fits-all approaches [34, 36]. These considerations directed the approach of the emerging collaboration between Indigenous and non-Indigenous co-researchers described below, namely in its participatory methodology, Indigenous-defined methods, and the involvement of representatives from varying Indigenous Australian cultures and localities.

Menstruation is considered private ‘women’s business’ in many Aboriginal and Torres Strait Islander cultures, making it a particularly sensitive topic to discuss. While acknowledging the potential social and cultural risks of discussing a women’s business issue in a research setting, the secrecy associated with menstruation may be one of the factors preventing women and girls from living comfortably and without shame every day of the month [2]. Acknowledgement of the tension between the significance of understanding how the lives of girls and women can be improved while honouring these cultural considerations is paramount. The potential distal benefits of improving MHH are long-reaching, including increased confidence, higher educational attainment, and ultimately, improved quality of life over the life course [19, 39, 40].

### Menstrual health and hygiene in Aboriginal and Torres Strait Islander populations

In 2017 the Central Australian Youth Link Up Service (CAYLUS) published educational material about MHH [41] for girls in remote Indigenous Australian communities after identifying many barriers to MHH faced by girls in the communities where they work. Meanwhile, The University of Queensland (UQ) and WaterAid Australia were conducting a study of WASH in remote Indigenous communities, which introduced researchers to the work CAYLUS had been doing. The final report from UQ and WaterAid Australia outlined anecdotal evidence indicating that MHH barriers exist for girls living in various rural and remote Indigenous Australian communities [20, 21, 41]. In that study, 17 qualitative interviews on WASH in remote Indigenous Australian communities were conducted among four Indigenous and 13 non-Indigenous stakeholders (government (*n* = 6) and non-government organisations (*n* = 2), research (*n* = 3), and utilities representatives (*n* = 2) representing organisations that provide WASH services to remote communities across four mainland Australian states and territories) [20]. Semi-structured interviews lasted approximately 45 min and captured perceptions of drinking water, wastewater management, and access to hygiene services among community members living in remote locations [20]. Without prompting, four interviewees explicitly described barriers to menstrual hygiene when speaking about hygiene priorities, For example, one interviewee stated:

*Mothers and grandmothers have said that girls are missing school when they have their periods … because they don't want to change [pads] at school … [at schools] often there's no soap, … there's often no rubbish bins or there's one rubbish bin outside the toilet which is really embarrassing to use. In terms of the infrastructure that I can put in place to help girls, it's rubbish bins, it's soap, it's running water and toilets that flush, and privacy* (Indigenous organisation representative #3).

Challenges described by interviewees in unprompted responses are described in quotes from the interviewees:

- **For some groups, Western cultural ‘shame’ prevents discussion:** ‘*There’s a lot of shame around it … [Also,] traditional forms of learning [aren’t] necessarily functioning within families for everything … Traditionally, it’s a grandmother’s role, …but a lot of grandmothers experienced mission times where there was very strong puritan Christian values around your body, which meant you don’t talk about it’* (Indigenous organisation representative #3).
- **MHM is a low priority in health education**: *‘there’s such a demand on every single [health education] resource available and everything is under-resourced. If you’re dealing with someone’s diabetes and if you’re dealing with chronic illness, [then] menstruation is not [seen as] a sickness … [MHM education] is missing in the [remote] regions and it is a serious concern that has an impact on girls’ and women’s lives. … [including] being able to go to school’* (Indigenous organisation representative #3).
- **Buying MHM products can be embarrassing and expensive:**
  *People aren’t going to the shop and buy it, because they’re tiny places and people will know that you’ve bought it because you’re menstruating … There’s sort of a bit of … stigmatising or feeling ashamed* (Research representative #2).
- **Alternatives are used as MHM products:** ‘*underwear is another thing that can be used and flushed down … the local plumbing services say there are a lot of problems with not just tampons being flushed, but various pieces of clothing’* (Indigenous organisation representative #3).

The responses from these interviewees mirrored published research outcomes from LMIC contexts, which identify lack of products, privacy, pain management resources, puberty education, and access to functioning health hardware as key aspects of managing menstruation [6, 42–44]. Reports from CALYUS also supported addressing educational factors that may be contributing to inequity in this space [41]. In response to these initial anecdotal findings [21], Aboriginal and Torres Strait Islander women from across the country contacted Dr. Nina Hall and further corroborated these reports. This included an invitation from the CEO of the National Aboriginal and Torres Strait Islander Women’s Alliance (NATSIWA), Sandra Creamer, to work in partnership to explore the state of MHH among Indigenous Australian communities [45].

## Working in partnership: the yarning circle

In this section we draw on insights from a partnership formed between a group of 12 Aboriginal and Torres Strait Islander women, some of whom are researchers, from urban, rural and remote areas, and eight non-Indigenous co-researchers and practitioners working on MHH and public health both within Australia and abroad. Together the group convened a participatory [29, 46], Indigenous-led yarning circle [47, 48] in Brisbane, Queensland, to identify barriers to MHH in Indigenous communities across Australia, and discuss opportunities for addressing such barriers [45]. The full-day session took place in March 2018.

Focused on maximising Indigenous Australian women’s participation, power and control in the process, a participatory approach was foregrounded as an iterative, reflexive means for non-Indigenous researchers to work *with* Indigenous partners [29, 35, 46, 49]. The participatory approach enabled co-researchers to acknowledge the historical dynamics previously stated, the value of local knowledge, and the mutual capacity building that can occur through equitable partnership [29, 50, 51]. In the words of Creamer, non-Indigenous university researchers would serve as the ‘hand’ and the Indigenous co-researchers as the ‘voice’ [Sandra Creamer to Nina Hall, personal communications, 1 Nov 2017].

The yarning circle method was specifically chosen to allow for an organic, culturally-appropriate discussion to occur and to draw on the insights of the group to provide directions for future efforts in Australia [47, 48]. NATSIWA deemed voice recording inappropriate for the setting, so a note taker (first author of this article) typed participants’ contributions on a laptop throughout the meeting. Conversations covered a range of issues around menstruation, including social taboos, infrastructure challenges, access to pubertal information, physical privacy, and barriers to purchasing and using products. A summary of action items from the yarning circle discussion was collectively compiled, drafted, and distributed to all participants for feedback following the session [45].

## Lessons from the yarning circle

In alignment with findings from previous work [41, 52], the experiences and observations of the Indigenous Australian women present at the yarning circle supported the notion that menstruation is likely to present challenges for many Indigenous Australian girls and women in varying locations across remote, regional, and urban settings. Notably, the Indigenous Australian women attending the yarning circle were primarily from a mature age demographic, so the issues prioritised by the group may differ from topics that would be explored by younger girls who are currently experiencing menstrual cycles- and that these younger and current perspectives of their experiences should ideally be heard in future research. The dialogue began with each attendee introducing and positioning themselves in the space, at which time one attendee underscored the importance of listening to younger generations’ stories to learn about their unique needs [45]. Though her message was not directly in relation to menstruation, the acknowledgement highlighted that the yarning circle attendees’ observations of younger generations’ menstrual experiences would offer rich insights, but that listening to and working with girls from their communities would be required to adequately understand their circumstances. However, attendees expressed that the stigma, secrecy, and shame involved with discussing menstruation and bleeding may prevent older women from exploring those issues with young people [45]. Additionally, a lack of contemporary, comprehensive puberty education that includes information on menstruation and menstrual hygiene was also suggested as a barrier [41]. Yarning circle attendees agreed that culturally-sensitive puberty education provision was needed for girls and boys, emphasising that MHH content and material must proceed with sensitivity, but should be made available to all genders. Outsider access to, or public dissemination of, traditional knowledge or practices was considered inappropriate and unnecessary for the development of improved menstrual education resources.

Other identified barriers included difficulties in storing and transporting products as a result of overcrowded housing, living across multiple residences, and lack of privacy for safe keeping of personal items. Logistical challenges related to the use of menstrual products discussed during the yarning circle include secrecy around purchasing sanitary items, high financial cost, sufficient female-friendly infrastructure (e.g., working toilets, accessible disposal facilities, privacy) [53, 54], and seasonal disruptions in supply due to weather. Attendees also noted the compounded challenges present for Indigenous Australian peoples among specific demographics such as boarding school students, young mothers, and homeless populations [45].

Following discussion of barriers, the yarning circle explored options for addressing the barriers that had been identified. MHH action areas were summarised and voted upon to prioritise next steps, summarised in the meeting report [45]. Each of the following actions were deemed necessary by the group, presented in the order prioritised by attendees (with the first actions receiving the most votes): 1) establish community-controlled research and evaluation; 2) ensure ongoing product distribution (while affordability and access are being improved); 3) build awareness and provide community-designed education regarding puberty and women’s reproductive health; 4) lobby for menstrual equity through government and community; and 5) engage with Indigenous Australian women in leading roles to share messages around menstrual health. There appear to be significant opportunities to begin implementing these actions through partnerships between Indigenous Australian peoples and communities, researchers, NGO officers, and policymakers [45].

## Directions for MHH research, advocacy, and support in Australia

The initial WASH assessment and subsequent yarning circle have received attention from NGOs and government and have resulted in a number of tangible outcomes occurring as implementations of the yarning circle priorities. For example, attendees have been invited to present at conferences, have coordinated donations of sanitary products for remote communities, and have secured funding to develop a pilot project in remote Queensland. Government has also shown interest in recent MHH efforts in Australia. Prior to the yarning circle, a motion was introduced to the Federal Senate to reduce MHH barriers for girls and women in remote communities [55], which cited the anecdotal research that initiated the yarning circle [21]. The report from the yarning circle [45] was submitted to the 2018 consultation on Indigenous Australian women’s voices detailing their intended future vision (Wiyi Yani U Thangani), conducted by the Aboriginal and Torres Strait Islander Social Justice Commissioner at the Australian Human Rights Commission [56].

The need for community and evidence-informed MHH approaches in Australia is held in tension with policy and practice focused on implementing interventions. Discussion from the authors’ previous research and the yarning circle suggests that to positively affect change, any research conducted must be Indigenous-led to ensure that actions are sensitive to community needs and lived experiences [31, 34, 57] and well-received by those who are impacted [58]. In considering MHH in the Australian context, researchers and policy makers must learn from past mistakes and avoid rushed implementation of programs that are not evidence-based or seek to obtain data without providing reciprocal benefits to participants [34].

## Considerations for future research

Though the interested response from NGOs and government reflects growing recognition of women’s menstrual needs, the anecdotal nature of evidence to date is insufficient to inform effective programs or policies. Introducing policies or significant resource outlays without research evidence increases likelihood of sub-optimal or even harmful outcomes [36, 57]. Consistent with past research around the world [6], discussion throughout the yarning circle identified privacy as a challenge for girls seeking to store or change menstrual products as well as experiencing discomfort with publicly purchasing products altogether. As such, simply reducing the cost of materials through the reduction of taxes may be insufficient to improve girls’ experiences. Additionally, provision of vending machines that provide free MHH products may improve access for girls while in school, but more evidence is needed to inform knowledge of the effectiveness and implementation of the strategy prior to scaling up. Drawing from the findings of other MHH research contexts, focus on product provision alone without attention to education and social stigma surrounding menstruation may be insufficient to improve women’s menstrual experiences and MHH across the life-course [10]. Insights from the yarning circle also aligned with the global call for improvements in access to, and quality of, female-friendly toilets [53, 54], particularly with disposal bins inside bathroom stalls in schools and homes. Inclusion of these aspects in the development and design of educational materials and interventions is critical.

Other considerations highlighted in the literature on effectively partnering with Indigenous Australian communities suggest that participatory action research (PAR) approaches can be useful [29, 35, 46], and discussions during the yarning circle highlighted that such approaches, which allow for community researchers to design and conduct research and action themselves, may be appropriate owing to the heterogeneity of norms and experiences among Indigenous Australians. For example, PAR approaches can include many qualitative and quantitative activities, e.g., communicating through stories and gaining perspectives from young people through in-depth discussion to quantitative surveys (one attendee expressed that health and safety questionnaires are common for some of the workplaces in her community, so a survey design may suit such a population). Similarly, depending on guidance from Indigenous partners, non-invasive data collection, such as leveraging existing school attendance and achievement records, may also be an option for evaluating interventions and assessing effectiveness without burdening communities.

## Conclusions

As global attention to MHH continues to increase, it is critically important to ensure that disparities in HICs are considered and addressed. Menstrual stigma and taboos have far reaching implications across populations, yet the experiences of those facing inequities in HICs have not yet been adequately heard. Developing initiatives that address menstruation from individual, familial, community, and societal levels will contribute to dismantling the barriers identified by researchers around the world. Further research is needed to understand the barriers to MHH among Indigenous Australian girls and women to then inform the development and evaluation of effective policy and practice. This research will necessarily involve co-researchers from communities, not overstep factors related to traditional practices, and ensure reciprocal benefits for communities and co-researchers. Interest from stakeholders to better understand MHH and corroborate its link to development and education are promising signs for continued momentum and future research. Community-driven initiatives focused on improving the wellbeing of girls are important steps toward improving the health of all communities by supporting the next generation of female leaders.

## Data Availability

Not applicable.

## Abbreviations

HICs: High Income Countries
LMICs: Low- and Middle-Income Countries
MHH: Menstrual health and hygiene
NATSIWA: National Aboriginal and Torres Strait Islander Women’s Alliance
NGOs: Non-government organisations
SDGs: Sustainable Development Goals
UQ: The University of Queensland
WASH: Water, sanitation, and hygiene

## Footnotes

1 This article uses the terms Aboriginal and Torres Strait Islander Peoples and Indigenous Australians interchangeably in reference to the original custodians of Australia [15, 16]. The term non-Indigenous Australian refers to people who have migrated to Australia since 1788 when British colonisation began [17].
